# All-Cause and Cause-Specific Mortality by SEER Stage in Gastric Cancer: A Nationwide Population-Based Cohort Study

**DOI:** 10.3390/jcm15093484

**Published:** 2026-05-02

**Authors:** Jihoon Hong, Mi Jin Oh, Bokyung Kim, Seunghan Lee, Yoon Jin Choi, Kyungdo Han, Soo-Jeong Cho

**Affiliations:** 1 Department of Internal Medicine and Liver Research Institute, Seoul National University College of Medicine, 101 Daehak-ro, Jongno-gu, Seoul 03080, Republic of Korea; 2 Department of Internal Medicine, Seoul Metropolitan Government Seoul National University Boramae Medical Center, Seoul 07061, Republic of Korea; 3 Center for Gastric Cancer, National Cancer Center, Goyang 10408, Republic of Korea; 4 Department of Statistics and Actuarial Science, Soongsil University, 369 Sangdo-ro, Dongjak-gu, Seoul 06978, Republic of Korea

**Keywords:** gastric cancer, cause of death, cardiovascular disease, lung diseases, SEER program

## Abstract

**Background:** Despite significant advances in diagnosis and treatment, gastric cancer remains a major global malignancy. This study aimed to evaluate the impact of Surveillance, Epidemiology, and End Result (SEER) stages on all-cause and cause-specific mortality in gastric cancer. **Methods:** This nationwide population-based cohort study analyzed data from the Cancer Public Library Database (CPLD). Patients aged ≥ 30 years diagnosed with gastric cancer between 2012 and 2019 were followed up until 31 December 2020. Cox proportional hazards models and Fine–Gray models were used to compare the risk of all-cause and cause-specific mortality based on SEER stages. The Kaplan–Meier method and cumulative incidence functions were applied to analyze cumulative incidences of all-cause and cause-specific mortality. Statistical significance was assessed using the log-rank test and Gray’s test. Additionally, a subgroup analysis was performed. **Results:** Among 218,491 individuals, 59,952 died during a median follow-up of 3.62 years. Compared with the localized stage, the risk of all-cause mortality was 4.31 and 24.73 times higher in patients with the regional and distant stages, respectively, after adjusting for sex, age, income, residential area, and comorbidities. The regional stage was associated with an 8.70-, 6.08-, 1.28-, and 1.43-fold higher risk of stomach cancer death, cancer death, cardiovascular death, and respiratory death, respectively. The distant stage was associated with 51.67-, 35.97-, 1.74, and 1.54-fold higher risk of stomach cancer death, cancer death, cardiovascular death, and respiratory death, respectively. **Conclusions:** Higher SEER stage in gastric cancer is associated with an increased risk of all-cause mortality, gastric cancer-specific mortality, overall cancer mortality, cardiovascular disease-related mortality, and respiratory disease-related mortality. Notably, cardiopulmonary mortality increased with advancing SEER stage, particularly among younger patients, underscoring the need for vigilant monitoring.

## 1. Introduction

Despite advances in diagnostic techniques and therapeutic strategies, gastric cancer remains one of the most prevalent and lethal malignancies worldwide [1]. According to global cancer statistics, gastric cancer ranked fifth worldwide in both incidence and cancer-related mortality in 2022 [2]. Gastric cancer has a particularly high incidence in East Asia, underscoring its public health importance in these countries [3]. In 2021, the age-standardized incidence of gastric cancer in Korea was 27.5 per 100,000 population, ranking fourth after thyroid, colorectal, and lung cancers. Furthermore, the age-standardized mortality was 5.9 per 100,000 population, also ranking fourth after lung, liver, and colorectal cancers [4].

The Surveillance, Epidemiology, and End Result (SEER) staging system—a cancer staging classification developed by the United States National Cancer Institute [5,6]—classifies cancer into three major stages: localized, regional, and distant [6]. Each SEER stage was defined as follows: localized, confined to the stomach; regional, involving adjacent structures or regional lymph nodes; and distant, indicating metastatic disease. The system classifies the extent of cancer progression in a simple and consistent manner, enabling evaluation and comparison of cancer incidence, treatment outcomes, and survival rates. In Korea, the Korea Central Cancer Registry (KCCR) has reported the SEER stage for specific cancers since 2006 [4,7].

As the prognosis of gastric cancer improves, it has become important for patients with gastric cancer to identify and prevent not only gastric cancer-related death but also non-gastric cancer-related death. This study aimed to analyze all-cause and cause-specific death according to SEER stages in patients with gastric cancer.

## 2. Materials and Methods

### 2.1. Data Source

This nationwide population-based cohort study utilized data from the Cancer Public Library Database (CPLD) of the Korean Clinical Data Utilization for Research Excellence project (K-CURE). The CPLD integrates four major population-based public data sources in Korea: the Korea National Cancer Incidence Database within the KCCR, cause-of-death data from Statistics Korea, National Health Information Database from the National Health Insurance Service, and National Health Insurance Research Database from the Health Insurance Review & Assessment Service. CPLD includes information about cancer registration, death, demographics, medical claims, general health checkups, and national cancer screening of 1,983,499 patients newly diagnosed with cancer between 2012 and 2019 in Korea. Specifically, the cancer registration data includes age at diagnosis, diagnosis date, cancer topography based on the International Statistical Classification of Diseases and Related Health Problems, 10th Revision (ICD-10), cancer morphology classified by the International Classification of Diseases for Oncology, 3rd Edition, SEER stage, and treatment method. The CPLD was established in 2022 and is updated annually [8]. This study was exempted from review by the Institutional Review Board of Seoul National University Hospital (IRB No. 2412-006-1590).

### 2.2. Study Population

**Figure 1:**
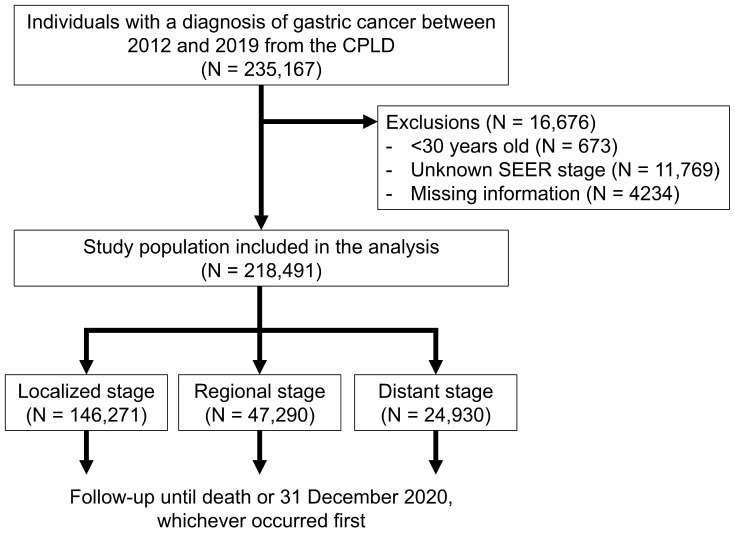
Flowchart of the study population selection. Abbreviations: CPLD, Cancer Public Library Database; SEER, Surveillance, Epidemiology, and End Results Program.

The study population selection process is illustrated in Figure 1. Between 2012 and 2019, 235,167 individuals diagnosed with gastric cancer (ICD-10 code C16) were identified in the CPLD. The final study population consisted of 218,491 participants after excluding individuals under 30 years of age, with unknown SEER stages, or with missing information. The index date was defined as the date of gastric cancer registration, and the follow-up end date was 31 December 2020. The study population was followed up until death or the end of the study period, whichever occurred first.

### 2.3. Study Outcomes

The primary outcome was the all-cause mortality. The secondary outcome was the cause-specific mortality, including gastric cancer-related death (ICD-10 code C16), cancer-related death (ICD-10 code C), cardiovascular disease-related death (ICD-10 code I), and respiratory disease-related death (ICD-10 code J). Cancer-related death was defined as overall cancer-related death, including gastric cancer-related death. Detailed definitions of these terms are summarized in Supplementary Table S1.

### 2.4. Statistical Analysis

Data were presented as mean and standard deviation for continuous variables or as numbers and percentages for categorical variables. Baseline characteristics were compared using one-way analysis of variance for continuous variables and the chi-square test for categorical variables. Hazard ratios (HRs) and 95% confidence intervals (CIs) were calculated using Cox proportional hazard models. The proportional hazards assumption was assessed using log-minus-log plots and Schoenfeld residuals. Subdistribution hazard ratios (sHRs) were estimated using Fine–Gray models to account for competing risks. Multivariable-adjusted analyses were conducted after adjusting for sex, age, income, residential area, diabetes, hypertension, and dyslipidemia. The Kaplan–Meier method was employed to compare cumulative incidence. Statistical significance was assessed using the log-rank test. Cumulative incidence functions were estimated to account for competing risks, and differences between groups were compared using Gray’s test. To evaluate the potential effect modifications by sex, age, income, residential area, diabetes, hypertension, and dyslipidemia, subgroup analyses and interaction tests were performed using the likelihood-ratio test. Statistical significance was set at two-tailed *p* < 0.05. Statistical analyses were performed using SAS software 9.4 (SAS Institute Inc., Cary, NC, USA) and R software 4.4.1.

## 3. Results

### 3.1. Baseline Characteristics

Baseline characteristics of the study population assessed on the index date are presented in Table 1. Among 218,491 individuals, 146,271, 47,290, and 24,930 were classified as having localized, regional, and distant stages, respectively. The mean age of the study population was 63.77 years, and 67.9% were male. Overall, 19.47% of the participants had a low income level and 43.01% resided in metropolitan areas. Among the participants, 20.98%, 45.95%, and 27.72% had diabetes, hypertension, and dyslipidemia, respectively. Initial treatment was defined as treatment administered to the primary site and metastatic sites of the cancer within four months of the initial diagnosis; in some cases, two or more treatments were administered. For initial treatment of gastric cancer, 82.13%, 19.92%, 0.77%, and 0.1% of the patients underwent surgery, chemotherapy, radiotherapy, and immunotherapy or hormonal therapy, respectively. The median follow-up duration was 3.62 years. Compared to patients with the regional and distant stages, patients with the localized stage were younger, more likely to be male, less likely to have low income, and more likely to reside in metropolitan areas. They also exhibited a lower prevalence of diabetes but a higher prevalence of dyslipidemia. The proportion of patients who underwent surgery as the initial treatment was higher, and the follow-up duration was longer in the patients with localized stage than in those with the regional and distant stages. All of these differences were statistically significant.

A comparison of baseline characteristics between the study population and the group of patients excluded from the study population due to unknown SEER stage is summarized in Supplementary Table S2. The group with unknown SEER stage was older and had a higher proportion of non-metropolitan residents compared to the study population. They also had higher rates of death and cause-specific death, resulting in a shorter follow-up duration. These differences were statistically significant.

### 3.2. Risk of All-Cause Mortality According to the SEER Stage

The all-cause mortality according to SEER stage is summarized in Table 2. Of the total study population, 59,952 individuals died during a median follow-up duration of 3.62 years. Specifically, 17,340, 19,888, and 22,724 individuals with localized, regional, and distant stages died, with an incidence rate of 26.09, 119.61, and 810.87 per 1000 person-years, respectively. In all Cox proportional hazards models—unadjusted (model 1), adjusted for sex and age (model 2), sex, age, income, and residential area (model 3), and fully adjusted for sex, age, income, residential area, and comorbidities (model 4)—a higher SEER stage was consistently associated with an increased risk of all-cause mortality. Initial treatment modality is generally determined by SEER stage and may act as a mediator in the relationship between SEER stage and outcomes; therefore, it was not included in the adjustment to avoid the risk of overadjustment. Compared with the localized stage, the risk of all-cause mortality was 4.31 and 24.73 times higher in patients with the regional and distant stages, respectively, in the fully adjusted model (regional stage: adjusted HR [aHR], 4.31; 95% CI, 4.22–4.40; distant stage: aHR, 24.73; 95% CI, 24.22–25.26).

### 3.3. Risk of Cause-Specific Mortality According to the SEER Stage

Cause-specific mortality according to SEER stage is presented in Table 3. Compared with the localized stage, regional and distant stages were associated with 8.70- and 51.67-fold higher risk of stomach cancer deaths, respectively, after adjusting for sex, age, income, residential area, and comorbidities (regional stage: aHR, 8.70; 95% CI, 8.46–8.94; distant stage: aHR, 51.67; 95% CI, 50.24–53.14). Compared with the localized stage, regional and distant stages were associated with a 6.08- and 35.97-fold higher risk of cancer deaths, respectively, in model 4 (regional stage: aHR, 6.08; 95% CI, 5.94–6.23; distant stage: aHR, 35.97; 95% CI, 35.11–36.85). Compared with the localized stage, regional and distant stages were associated with 1.28- and 1.74-fold higher risk of cardiovascular deaths, respectively, in the fully adjusted model (regional stage: aHR, 1.28; 95% CI, 1.16–1.41; distant stage: aHR, 1.74; 95% CI, 1.43–2.12). Compared with the localized stage, regional and distant stages were associated with a 1.43- and 1.54-fold higher risk of respiratory deaths, respectively, after adjusting for sex, age, income, residential area, and comorbidities (regional stage: aHR, 1.43; 95% CI, 1.28–1.59; distant stage: aHR, 1.54; 95% CI, 1.19–2.00).

### 3.4. Cumulative Incidence According to the SEER Stage

**Figure 2:**
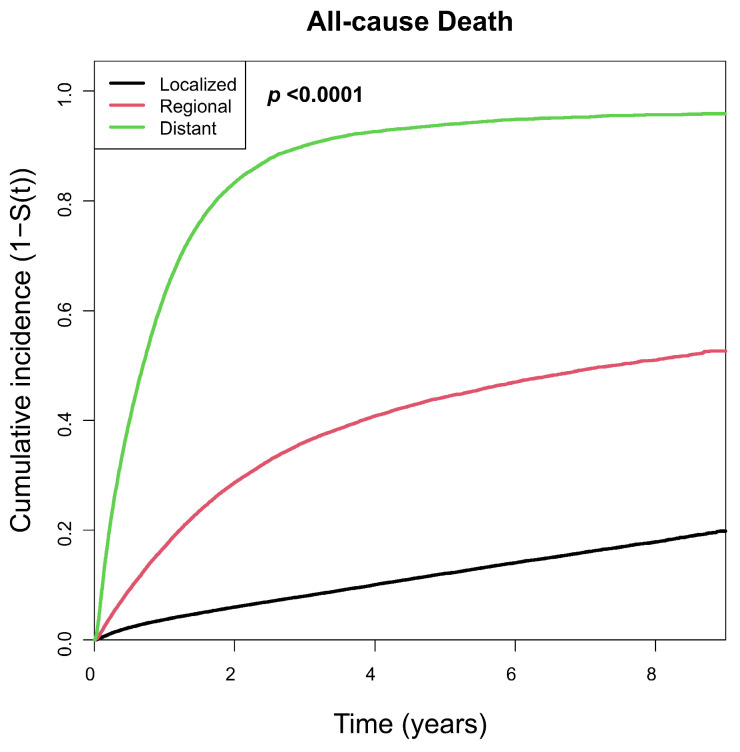
Kaplan–Meier-based cumulative incidence of all-cause mortality according to the SEER stage. The black, red, and green lines represent the Kaplan-Meir-based cumulative incidence rates for localized, regional, and distant stages, respectively. Abbreviation: SEER—Surveillance, Epidemiology, and End Results Program.

**Figure 3:**
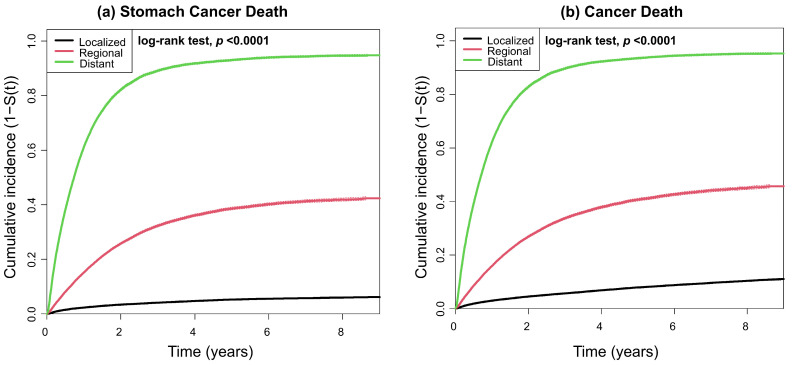

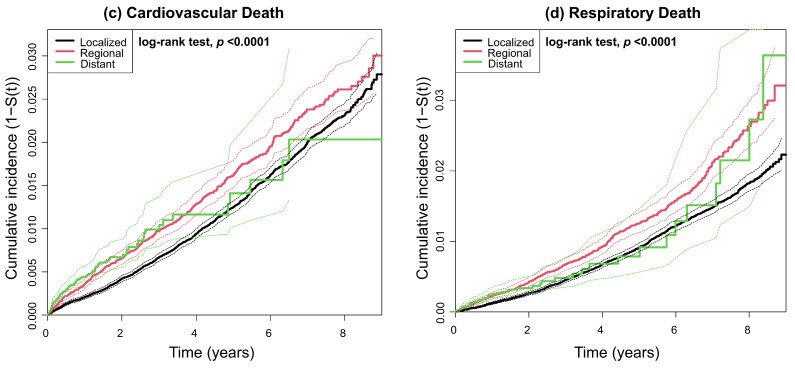
Kaplan–Meier-based cumulative incidence of cause-specific mortality according to the SEER stage. (**a**–**d**) The black, red, and green lines represent the Kaplan-Meir-based cumulative incidence rates for localized, regional, and distant stages, respectively. The dashed lines indicate confidence intervals. Abbreviation: SEER—Surveillance, Epidemiology, and End Results Program.

The Kaplan–Meier-based cumulative incidences of all-cause and cause-specific deaths are shown in Figure 2 and Figure 3. The cumulative incidence of all-cause death increased significantly with higher SEER stage (Figure 2). Similarly, the cumulative incidence of stomach cancer and cancer deaths significantly increased with higher SEER stage (Figure 3a,b). Although the Kaplan–Meier-based cumulative incidence of cardiovascular and respiratory deaths differed significantly across stages, no clear trend was observed (Figure 3c,d).

**Figure 4:**
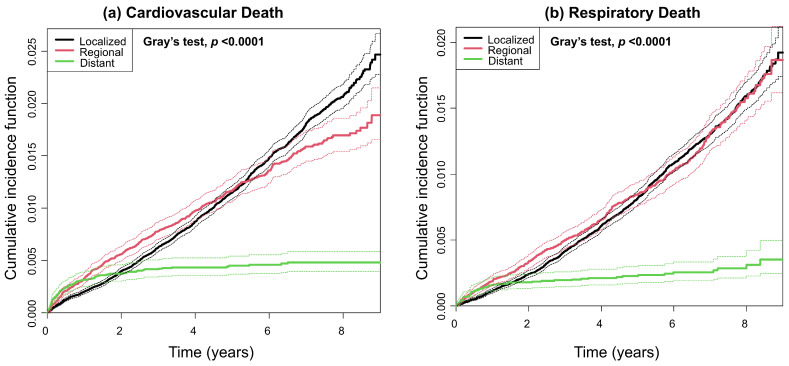
Cumulative incidence function of cardiovascular and respiratory death according to the SEER stage. (**a**,**b**) The black, red, and green lines represent the cumulative incidence function for localized, regional, and distant stages, respectively. The dashed lines indicate confidence intervals. Abbreviation: SEER—Surveillance, Epidemiology, and End Results Program.

Because cardiovascular and respiratory deaths were relatively infrequent and subject to substantial competing risks, cumulative incidence functions are presented in Figure 4. Both cardiovascular and respiratory deaths showed lower cumulative incidence in the distant stage than in the localized and regional stages, and these differences were statistically significant according to Gray’s test (Figure 4a,b). This finding can be interpreted as reflecting the high burden of stomach cancer-related mortality in the distant stage, which limits the occurrence of cardiovascular and respiratory deaths.

### 3.5. Subgroup Analysis

The results of the subgroup analyses of all-cause mortality according to sex, age, income, residential area, and comorbidities are summarized in Table 4. The HRs were adjusted for sex, age, income, residential area, diabetes, hypertension, and dyslipidemia. Across all groups, a higher SEER stage was consistently associated with a significant increase in the risk of all-cause mortality. The effect of SEER stage on all-cause mortality was significantly more pronounced in females, younger individuals (aged 30–64 years), those with higher income, metropolitan residents, and individuals without diabetes, hypertension, or dyslipidemia (*p* for interaction < 0.05).

The results of the subgroup analysis of cause-specific mortality according to sex, age, income, residential area, and comorbidities are summarized in Supplementary Tables S3–S6. The effect of SEER stage on stomach cancer death was statistically more pronounced among males, younger individuals, those with a higher income, metropolitan residents, individuals without diabetes or hypertension, and those with dyslipidemia (*p* for interaction < 0.05). The impact of SEER stage on cancer death was significantly more pronounced among females, younger individuals, those with higher income, metropolitan residents, and individuals without diabetes or hypertension but with dyslipidemia (*p* for interaction < 0.05). The effect of the SEER stage on cardiovascular and respiratory deaths was significantly more pronounced among younger individuals (*p* for interaction < 0.05), but not by sex, income, residential area, or comorbidities (*p* for interaction > 0.05).

## 4. Discussion

This nationwide population-based cohort study evaluated the association of SEER stages with all-cause and cause-specific mortality in patients with gastric cancer. The primary results demonstrated a strong association between an advanced SEER stage and increased all-cause mortality. As expected, we found that higher SEER stage was associated with higher all-cause mortality in patients with gastric cancer.

Our findings demonstrated that overall cancer mortality risk increases with advancing stage of gastric cancer. This increased risk may be primarily attributable to the heightened mortality risk of gastric cancer itself. However, with advancements in diagnosis and treatment to improve the survival rates of patients with gastric cancer, the prevention of deaths caused by second primary malignancies (SPMs) among gastric cancer survivors has become increasingly important. Patients with gastric cancer exhibit an increased risk of developing SPMs compared with the general population [9,10]. Chen et al. identified age ≥ 70 years, male sex, diabetes mellitus, chronic obstructive pulmonary disease (COPD), liver cirrhosis, chemotherapy, and radiotherapy as independent risk factors for SPMs in patients with gastric cancer [9]. Regular follow-up examinations are necessary to monitor not only gastric cancer recurrence but also the development of metachronous SPMs in gastric cancer survivors. Further research is also needed to determine more common types of metachronous SPMs observed among gastric cancer survivors.

Advancements in early diagnosis and treatment have led to steady improvements in the survival rates of gastric cancer [11,12]. Consequently, addressing non-gastric cancer-related mortality in these patients has become increasingly important. Several previous studies have reported an increased risk of cardiovascular death in patients with cancer [13,14,15]. This increased risk may be attributable to the shared risk factors between cancer and cardiovascular disease, cancer-induced inflammation, and adverse cardiac effects associated with cancer treatments [16]. In particular, trastuzumab, which is commonly used in chemotherapy for HER2-positive gastric cancer, is well known to cause cardiomyopathy. Previous studies have also reported an elevated risk of cardiovascular mortality in patients with gastric cancer [17,18]. Furthermore, lifestyle modifications, such as smoking cessation or alcohol abstinence, are important for preventing the development of cardiovascular disease in patients who have undergone gastrectomy [19]. Although the cumulative incidence of cardiovascular death was low in the distant stage (Figure 4a, green line), likely due to the predominance of gastric cancer-related mortality, the adjusted hazard ratio for cardiovascular death increased with advancing stage. Therefore, greater efforts are needed to prevent cardiovascular death in gastric cancer survivors with more advanced stages. There is a need to establish long-term surveillance strategies in gastric cancer survivors to monitor not only cancer recurrence but also the potential development of cardiovascular disease.

In this study, higher-stage gastric cancer was associated with an increased risk of respiratory death. A limited number of previous studies have analyzed the risk of respiratory diseases among patients with gastric cancer. A study examining the causes of death in patients with metastatic cancer reported heart disease (32.4%), COPD (7.9%), cerebrovascular disease (6.1%), and infection (4.1%) as the leading non-cancer causes of death [20]. Similarly, a study analyzing the causes of death among gastric cancer survivors in the United States from 2000 to 2020 reported non-cancer causes of death in the following order: heart disease (30%), cerebrovascular disease (6%), and COPD (6%) [21]. In another study, patients with cancer exhibited a 2.17-fold higher standardized mortality ratio for COPD than the general population [22]. Smoking and other common risk factors for both cancer and COPD may contribute to this increased risk. Because this study included patients newly diagnosed with gastric cancer between 2012 and 2019, the proportion of patients receiving immunotherapy was very low, accounting for less than 1% even in the distant stage, and thus may not fully reflect current treatment patterns. Currently, immune checkpoint inhibitors are widely used in the treatment of gastric cancer, and their indications are expanding. However, these agents are known to cause immune-related adverse events, including pneumonitis. Therefore, the clinical importance of respiratory complications is expected to increase among gastric cancer survivors. Careful monitoring is warranted to prevent respiratory death, especially in patients with advanced-stage gastric cancer.

The subgroup analysis in this study revealed a more pronounced effect of disease stage on all-cause mortality in females, younger individuals, those with higher income, metropolitan residents, and individuals without diabetes, hypertension, or dyslipidemia. This may reflect that in relatively healthier or socioeconomically advantaged populations, the effect of disease stage on mortality is more directly observable, whereas in patients with multiple comorbidities or lower socioeconomic status, other competing risks may attenuate this association. When analyzing non-cancer mortality, including cardiovascular and respiratory mortality, according to SEER stage, age was the only variable that demonstrated a statistically significant interaction. The effect of gastric cancer stage on both cardiovascular and respiratory deaths was more pronounced in the younger age group. In this group, the regional stage was associated with a 1.78-fold increased risk of cardiovascular death and a 1.46-fold increased risk of respiratory death, whereas the distant stage was associated with a 3.93-fold and 3.80-fold higher risk of cardiovascular and respiratory deaths, respectively. These findings indicate an increased risk of cardiovascular and respiratory deaths in younger patients with advanced-stage gastric cancer. Therefore, in addition to cancer treatment, preventing cardiopulmonary mortality through vigilant monitoring and timely intervention is essential in this group. However, given the exploratory nature of the subgroup analyses and the lack of adjustment for multiple comparisons, the potential for type I error should be acknowledged.

In this study, we employed Cox proportional hazards models to estimate cause-specific hazard ratios for cardiovascular and respiratory mortality according to SEER stage in patients with gastric cancer. However, because these outcomes are subject to a strong competing risk—namely, stomach cancer-related death—we additionally performed Fine–Gray subdistribution hazard regression to appropriately account for this competing risk structure. The results are summarized in Supplementary Table S7. In the Fine–Gray model, patients with regional and distant stage disease showed significantly lower subdistribution hazard ratios for cardiovascular mortality (sHR: 0.83 and 0.31, respectively) and respiratory mortality (sHR: 0.85 and 0.21, respectively) compared with those with localized stage disease. The opposing directions of association observed between the Fine–Gray and Cox models are expected and reflect the strong influence of stomach cancer-related death as a dominant competing event. Specifically, although patients with regional and distant stage disease exhibit higher cause-specific hazards of cardiovascular and respiratory death, the large number of stomach cancer-related deaths in these groups substantially attenuates the cumulative probability of cardiovascular and respiratory mortality at the population level. This pattern is particularly evident in distant-stage patients, in whom the markedly reduced cumulative incidence of cardiovascular and respiratory death is visually corroborated by the lower cumulative incidence function curve shown in Figure 4a,b (green line).

As a sensitivity analysis, patients who died within 90 days of gastric cancer diagnosis were excluded, and hazard ratios for all-cause mortality, stomach cancer-specific mortality, overall cancer mortality, cardiovascular mortality, and respiratory mortality according to SEER stage are summarized in Supplementary Table S8. The results were materially unchanged compared with the primary analysis, with higher SEER stage consistently associated with increased risks across both primary and secondary outcomes, thereby supporting the robustness of the findings.

Because smoking, alcohol use, and body mass index (BMI) are closely associated with cardiovascular and respiratory mortality, we conducted a sensitivity analysis restricted to patients with available data on these variables; the results are summarized in Supplementary Table S9. A total of 98,669 patients who underwent a health examination within 2 years of their initial gastric cancer diagnosis and had available information on smoking, alcohol use, and BMI were included in this analysis. After additional adjustment for smoking, alcohol use, and BMI, cardiovascular mortality remained higher with increasing SEER stage (aHRs: 1.34 and 1.98 for regional and distant stages, respectively). For respiratory mortality, a statistically significant 1.52-fold increased risk was observed only in patients with regional-stage disease.

The strength of this study lies in its design as a nationwide population-based study characterized by a large population representative of the Korean population. Notably, CPLD includes 96.7% of all cancer cases in Korea from 2012 to 2019 [8]. Furthermore, the long follow-up duration is another strength, achieving sufficient statistical power to identify differences in all-cause and cause-specific mortality according to the SEER stage for gastric cancer. Moreover, the diverse information from the CPLD enabled adjustments for key socioeconomic status factors, such as income and residential area.

Nevertheless, this study also has some limitations. First, owing to the retrospective nature of the study, we could not control for confounders such as smoking [23], alcohol consumption [24], obesity [23], physical inactivity [25], poor dietary habits [26], and comorbidities, including impaired fasting glucose [27,28], which are the known risk factors for both gastric cancer and all-cause mortality. Sensitivity analyses were conducted in a subset of participants with available data on smoking, alcohol use, and BMI (Supplementary Table S9); however, these variables were not ascertainable for the entire cohort. In addition, adjustment for Helicobacter pylori infection and eradication status—established strong risk factors for gastric cancer—was not feasible. Furthermore, we were unable to adjust for potential confounders such as the specific type of surgery or chemotherapy regimen. In particular, certain chemotherapeutic agents, such as trastuzumab and fluorouracil, are associated with cardiotoxicity, whereas other chemotherapeutic agents, such as immune checkpoint inhibitors, can lead to pulmonary adverse events. Therefore, assessing the use of these agents is crucial when analyzing the risk of cardiovascular and respiratory mortality. Second, although an increased risk of cardiopulmonary death was observed in patients with higher-stage gastric cancer, a definitive causal relationship between stage and these outcomes could not be established, and the underlying mechanisms of this association remain unclear. Third, excluding patients with unknown SEER stage may have introduced selection bias. As shown in Supplementary Table S2, this group was older, had a higher proportion of non-metropolitan residents, and exhibited higher rates of all-cause and cause-specific mortality, resulting in a shorter median follow-up duration. This heterogeneity between this group and the study population suggests the possibility of selection bias. Finally, as this was a nationwide population-based study conducted in Korea, the findings may not be generalized to Western populations.

Future studies incorporating detailed treatment and clinical variables, including the type of chemotherapeutic agents, use of immune checkpoint inhibitors, smoking status, alcohol use, BMI, and Helicobacter pylori infection status, are needed to adequately control for important confounders. In addition, further research is warranted to identify additional factors beyond gastric cancer stage that contribute to an increased risk of cardiopulmonary mortality, which may enable more refined risk stratification. Ultimately, efforts should focus on establishing tailored cardiopulmonary screening and management strategies for high-risk patients.

In conclusion, this nationwide population-based study demonstrated that all-cause mortality increases with higher SEER stage in patients with gastric cancer. Specifically, a higher SEER stage was associated with an increased risk of gastric cancer-specific death, overall cancer death, and cardiovascular and respiratory deaths. Notably, in the younger age group, the risk of cardiovascular and respiratory deaths increased substantially with increasing SEER stages, highlighting the importance of close monitoring in preventing cardiopulmonary mortality in this population.

## Figures and Tables

**Table 1:** Baseline characteristics of the study population according to the SEER stage.

	Total (N = 218,491)	Localized (N = 146,271)	Regional (N = 47,290)	Distant (N = 24,930)	*p*-Value
Age, years (mean (SD))	63.77 (11.98)	63.49 (11.52)	64.46 (12.59)	64.09 (13.31)	<0.0001
Age, years (%)					<0.0001
30–64	110,749 (50.69)	75,643 (51.71)	22,699 (48.00)	12,407 (49.77)	
≥65	107,742 (49.31)	70,628 (48.29)	24,591 (52.00)	12,523 (50.23)	
Sex (%)					0.0003
Male	148,364 (67.9)	99,729 (68.18)	31,789 (67.22)	16,846 (67.57)	
Female	70,127 (32.1)	46,542 (31.82)	15,501 (32.78)	8084 (32.43)	
Income (%)					<0.0001
Low	42,546 (19.47)	27,073 (18.51)	9989 (21.12)	5484 (22)	
Middle	109,047 (49.91)	72,169 (49.34)	24,133 (51.03)	12,745 (51.12)	
High	66,898 (30.62)	47,029 (32.15)	13,168 (27.85)	6701 (26.88)	
Residential area (%)					0.0004
Metropolitan	93,978 (43.01)	63,329 (43.3)	20,001 (42.29)	10,648 (42.71)	
Non-metropolitan	124,513 (56.99)	82,942 (56.7)	27,289 (57.71)	14,282 (57.29)	
Comorbidity (%)					
Diabetes	45,849 (20.98)	30,316 (20.73)	10,233 (21.64)	5300 (21.26)	<0.0001
Hypertension	100,402 (45.95)	67,563 (46.19)	21,854 (46.21)	10,985 (44.06)	<0.0001
Dyslipidemia	60,567 (27.72)	43,841 (29.97)	11,564 (24.45)	5162 (20.71)	<0.0001
Initial treatment modality (%)					
Surgery	179,445 (82.13)	133,608 (91.34)	39,999 (84.58)	5838 (23.42)	<0.0001
Chemotherapy	43,530 (19.92)	7112 (4.86)	20,893 (44.18)	15,525 (62.27)	<0.0001
Radiation therapy	1684 (0.77)	188 (0.13)	620 (1.31)	876 (3.51)	<0.0001
Immunotherapy or Hormonal therapy	219 (0.1)	71 (0.05)	44 (0.09)	104 (0.42)	<0.0001
Follow-up duration, years (median (IQR))	3.62 (1.67–6.06)	4.41 (2.51–6.54)	2.93 (1.35–5.48)	0.70 (0.27–1.39)	<0.0001
Death (%)	59,952 (27.44)	17,340 (11.85)	19,888 (42.06)	22,724 (91.15)	<0.0001
Stomach cancer death	45,105 (20.64)	6938 (4.74)	16,515 (34.92)	21,652 (86.85)	<0.0001
Cancer death	50,681 (23.2)	10,733 (7.34)	17,689 (37.41)	22,259 (89.29)	<0.0001
Cardiovascular death	2432 (1.11)	1766 (1.21)	555 (1.17)	111 (0.45)	<0.0001
Respiratory death	1820 (0.83)	1306 (0.89)	452 (0.96)	62 (0.25)	<0.0001

SEER—surveillance, epidemiology, and end results; SD—standard deviation; IQR—interquartile range.

**Table 2:** All-cause mortality according to the SEER stage.

	No.	Events	Person-Years	IR ^a^	HR (95% CI)			
					Model 1 ^b^	Model 2 ^c^	Model 3 ^d^	Model 4 ^e^
All-Cause Death								
Localized	146,271	17,340	664,544	26.09	1 (reference)	1 (reference)	1 (reference)	1 (reference)
Regional	47,290	19,888	166,273	119.61	4.38 (4.29–4.47)	4.38 (4.30–4.47)	4.37 (4.28–4.46)	4.31 (4.22–4.40)
Distant	24,930	22,724	28,024	810.87	23.26 (22.78–23.75)	25.26 (24.73–25.79)	25.15 (24.63–25.68)	24.73 (24.22–25.26)

SEER—surveillance, epidemiology, and end results, HR—hazard ratio, CI—confidence interval, IR—incidence rate. ^a^ Incidence rate: Per 1000 person-years. ^b^ Model 1: Non-adjusted. ^c^ Model 2: Adjusted for sex and age. ^d^ Model 3: Adjusted for sex, age, income, and residential area. ^e^ Model 4: Adjusted for sex, age, income, residential area, diabetes, hypertension, and dyslipidemia.

**Table 3:** Cause-specific mortality according to the SEER stage.

	Events	Person-Years	IR ^a^	HR (95% CI)			
				Model 1 ^b^	Model 2 ^c^	Model 3 ^d^	Model 4 ^e^
Stomach Cancer Death							
Localized	6938	664,544	10.44	1 (reference)	1 (reference)	1 (reference)	1 (reference)
Regional	16,515	166,273	99.32	8.86 (8.61–9.11)	8.85 (8.61–9.11)	8.83 (8.58–9.08)	8.70 (8.46–8.94)
Distant	21,652	28,024	772.62	49.15 (47.80–50.54)	52.89 (51.43–54.39)	52.73 (51.27–54.22)	51.67 (50.24–53.14)
Cancer Death							
Localized	10,733	664,544	16.15	1 (reference)	1 (reference)	1 (reference)	1 (reference)
Regional	17,689	166,273	106.39	6.19 (6.05–6.34)	6.20 (6.05–6.35)	6.18 (6.03–6.33)	6.08 (5.94–6.23)
Distant	22,259	28,024	794.28	34.15 (33.34–34.98)	36.85 (35.97–37.74)	36.72 (35.85–37.62)	35.97 (35.11–36.85)
Cardiovascular Death							
Localized	1766	664,544	2.66	1 (reference)	1 (reference)	1 (reference)	1 (reference)
Regional	555	166,273	3.34	1.27 (1.16–1.40)	1.26 (1.15–1.39)	1.26 (1.14–1.39)	1.28 (1.16–1.41)
Distant	111	28,024	3.96	1.58 (1.30–1.92)	1.70 (1.39–2.07)	1.69 (1.39–2.06)	1.74 (1.43–2.12)
Respiratory Death							
Localized	1306	664,544	1.97	1 (reference)	1 (reference)	1 (reference)	1 (reference)
Regional	452	166,273	2.72	1.43 (1.29–1.59)	1.44 (1.30–1.61)	1.44 (1.29–1.60)	1.43 (1.28–1.59)
Distant	62	28,024	2.21	1.41 (1.09–1.83)	1.56 (1.20–2.03)	1.55 (1.19–2.01)	1.54 (1.19–2.00)

SEER—surveillance, epidemiology, and end results, HR—hazard ratio, CI—confidence interval. ^a^ Incidence rate: Per 1000 person-years. ^b^ Model 1: Non-adjusted. ^c^ Model 2: Adjusted for sex and age. ^d^ Model 3: Adjusted for sex, age, income, and residential area. ^e^ Model 4: Adjusted for sex, age, income, residential area, diabetes, hypertension, and dyslipidemia

**Table 4:** Subgroup analysis of all-cause mortality by sex, age, income, residential area, and comorbidities.

	aHR (95% CI) ^a^ for All-Cause Mortality			*p* for Interaction
	Localized	Regional	Distant	
Sex				<0.0001
Male	1 (reference)	4.11 (4.01–4.21)	22.69 (22.14–23.25)	
Female	1 (reference)	4.84 (4.66–5.03)	30.24 (29.14–31.38)	
Age				<0.0001
30–64	1 (reference)	8.32 (7.98–8.68)	61.25 (58.84–63.75)	
≥65	1 (reference)	3.41 (3.33–3.49)	16.04 (15.63–16.45)	
Income				<0.0001
Low	1 (reference)	3.83 (3.67–3.99)	20.31 (19.48–21.18)	
Middle and High	1 (reference)	4.46 (4.36–4.57)	26.21 (25.60–26.84)	
Residential area				<0.0001
Metropolitan	1 (reference)	4.40 (4.26–4.54)	26.67 (25.85–27.53)	
Non-metropolitan	1 (reference)	4.25 (4.14–4.36)	23.42 (22.80–24.06)	
Diabetes				<0.0001
No	1 (reference)	4.67 (4.56–4.79)	28.09 (27.41–28.77)	
Yes	1 (reference)	3.50 (3.36–3.63)	17.20 (16.53–17.89)	
Hypertension				<0.0001
No	1 (reference)	5.49 (5.32–5.67)	34.29 (33.25–35.36)	
Yes	1 (reference)	3.60 (3.51–3.70)	18.72 (18.20–19.25)	
Dyslipidemia				<0.0001
No	1 (reference)	4.35 (4.25–4.46)	25.53 (24.92–26.15)	
Yes	1 (reference)	4.23 (4.07–4.40)	22.47 (21.58–23.39)	

aHR—adjusted hazard ratio. CI—confidence interval. ^a^ Adjusted for sex, age, income, residential area, diabetes, hypertension, and dyslipidemia.

## Data Availability

This study utilized the sample database of the K-CURE Cancer Public Library, developed by the National Cancer Data Center at the National Cancer Center in Korea, as part of the K-CURE project led by the Ministry of Health and Welfare. The database includes data provided by Public Institutions Working Groups, including the Korea Central Cancer Registry, Health Insurance Review & Assessment Service, National Health Insurance Service, Statistics Korea, and the Korea Disease Control and Prevention Agency. Data analyzed in this study will be available to other researchers upon reasonable request.

## Supplementary Materials

The following supporting information can be downloaded at: https://www.mdpi.com/article/10.3390/jcm15093484/s1, Table S1: Definition of terms; Table S2: Baseline characteristics of the study population and the group with unknown SEER stage; Table S3: Subgroup analysis of stomach cancer death by sex, age, income, residential area, and comorbidities; Table S4: Subgroup analysis of overall cancer death by sex, age, income, residential area, and comorbidities; Table S5: Subgroup analysis of cardiovascular death by sex, age, income, residential area, and comorbidities; Table S6: Subgroup analysis of respiratory death by sex, age, income, residential area, and comorbidities; Table S7: Adjusted hazard ratios (aHR) and subdistribution hazard ratios (sHR) for all-cause and cause-specific mortality according to the SEER stage; Table S8: Sensitivity analysis excluding patients who died within 90 days of diagnosis: All-cause and cause-specific mortality according to the SEER stage; Table S9: Sensitivity analysis for additional adjustment for smoking, alcohol use, and body mass index: All-cause and cause-specific mortality according to the SEER stage.
