# Supplementary material for: All-Cause and Cause-Specific Mortality by SEER Stage in Gastric Cancer: A Nationwide Population-Based Cohort Study
Source: J Clin Med. 2026 May 2;15(9):3484. doi: 10.3390/jcm15093484 (PMC13164358; doi:10.3390/jcm15093484)
Supplement: Supplementary file 1 [file jcm-15-03484-s001.zip › jcm-4175146-supplementary.pdf]

Supplementary Table S1. Definition of terms

**Terms**	**Definition**
Gastric cancer-related death	ICD-10 code C16
Cancer-related death	ICD-10 code C (including C16 for gastric cancer-related death)
Cardiovascular disease-related death	ICD-10 code I
Respiratory disease-related death	ICD-10 code J
Initial treatment modality	Initial treatment administered within 4 months of diagnosis for either the primary tumor or metastatic sites
Low-income	Medical aid recipients or 10th-20th income percentile
Middle-income	30th-80th income percentile
High-income	90th-100th income percentile
Metropolitan	Seoul or other metropolitan areas
Non-metropolitan	All other regions
Diabetes	ICD-10 codes E11–E14 and on antidiabetic medications
Hypertension	ICD-10 codes I10-I13, I15 and on antihypertensive medications
Dyslipidemia	ICD-10 code E78 and on antidyslipidemic medications

ICD-10 International Statistical Classification of Diseases and Related Health Problems, 10th Revision

Supplementary Table S2. Baseline characteristics of the study population and the group with unknown SEER stage

	Study population (N=218,491)	Unknown SEER stage (N=11,669)	*p*-value
Age, years (mean (SD))	63.77 (11.98)	71.08 (12.63)	<0.0001
Sex (%)			<0.0001
Male	148,364 (67.9)	7104 (60.9)	
Female	70,127 (32.1)	4565 (39.1)	
Income (%)			<0.0001
Low	42,546 (19.47)	2725 (23.35)	
Middle	109,047 (49.91)	5235 (44.86)	
High	66,898 (30.62)	3709 (31.79)	
Residential area (%)			<0.0001
Metropolitan	93,978 (43.01)	4452 (38.15)	
Non-metropolitan	124,513 (56.99)	7217 (61.85)	
Comorbidity (%)			
Diabetes	45,849 (20.98)	2719 (23.3)	<0.0001
Hypertension	100,402 (45.95)	6150 (52.7)	<0.0001
Dyslipidemia	60,567 (27.72)	3214 (27.54)	0.6763
Initial treatment modality (%)			
Surgery	179,445 (82.13)	2525 (21.64)	<0.0001
Chemotherapy	43,530 (19.92)	892 (7.64)	<0.0001
Radiation therapy	1684 (0.77)	121 (1.04)	0.0015
Immunotherapy or Hormonal therapy	219 (0.1)	3 (0.03)	0.0115
Follow-up duration, years (median (IQR))	3.62 (1.67-6.06)	1.75 (0.51-4.34)	<0.0001
Death (%)	59,952 (27.44)	6730 (57.67)	<0.0001
Stomach cancer death	45,105 (20.64)	5199 (44.55)	<0.0001
Cancer death	50,681 (23.2)	5803 (49.73)	<0.0001
Cardiovascular death	2432 (1.11)	255 (2.19)	<0.0001
Respiratory death	1820 (0.83)	172 (1.47)	<0.0001

SEER surveillance, epidemiology, and end results, SD standard deviation, IQR interquartile range

Supplementary Table S3. Subgroup analysis of stomach cancer death by sex, age, income, residential area, and comorbidities

	aHR (95% CI)[a] for stomach cancer death			*p* for interaction
	Localized	Regional	Distant	
Sex				<0.0001
Male	1 (reference)	9.23 (8.91-9.56)	53.86 (52.03-55.75)	
Female	1 (reference)	7.78 (7.43-8.16)	47.88 (45.73-50.13)	
Age				<0.0001
30-64	1 (reference)	20.42 (19.16-21.77)	145.38 (136.62-154.71)	
≥65	1 (reference)	6.44 (6.24-6.65)	32.84 (31.78-33.93)	
Income				<0.0001
Low	1 (reference)	7.25 (6.85-7.67)	39.92 (37.77-42.19)	
Middle and High	1 (reference)	9.18 (8.89-9.49)	55.78 (54.02-57.59)	
Residential area				<0.0001
Metropolitan	1 (reference)	8.94 (8.56-9.34)	55.56 (53.23-57.99)	
Non-metropolitan	1 (reference)	8.52 (8.22-8.84)	49.03 (47.29-50.83)	
Diabetes				<0.0001
No	1 (reference)	9.12 (8.83-9.43)	55.85 (54.08-57.69)	
Yes	1 (reference)	7.56 (7.16-7.98)	40.51 (38.38-42.75)	
Hypertension				<0.0001
No	1 (reference)	10.81 (10.36-11.29)	67.77 (64.98-70.67)	
Yes	1 (reference)	7.30 (7.03-7.58)	40.65 (39.17-42.19)	
Dyslipidemia				0.0018
No	1 (reference)	8.44 (8.17-8.72)	50.54 (48.94-52.19)	
Yes	1 (reference)	9.49 (8.97-10.03)	54.86 (51.91-57.97)	

aHR adjusted hazard ratio, CI confidence interval

[a] Adjusted for age, income, residential area, diabetes, hypertension, and dyslipidemia

Supplementary Table S4. Subgroup analysis of overall cancer death by sex, age, income, residential area, and comorbidities

	aHR (95% CI)[a] for overall cancer death			*p* for interaction
	Localized	Regional	Distant	
Sex				<0.0001
Male	1 (reference)	5.92 (5.75-6.09)	34.03 (33.07-35.02)	
Female	1 (reference)	6.48 (6.21-6.77)	40.61 (38.94-42.36)	
Age				<0.0001
30-64	1 (reference)	12.26 (11.66-12.90)	88.52 (84.32-92.92)	
≥65	1 (reference)	4.69 (4.56-4.83)	23.51 (22.84-24.20)	
Income				<0.0001
Low	1 (reference)	5.35 (5.10-5.63)	29.56 (28.16-31.03)	
Middle and High	1 (reference)	6.31 (6.14-6.49)	38.08 (37.05-39.13)	
Residential area				<0.0001
Metropolitan	1 (reference)	6.22 (5.99-6.46)	38.60 (37.22-40.04)	
Non-metropolitan	1 (reference)	5.98 (5.80-6.17)	34.19 (33.15-35.27)	
Diabetes				<0.0001
No	1 (reference)	6.49 (6.31-6.68)	39.64 (38.55-40.76)	
Yes	1 (reference)	5.08 (4.85-5.32)	26.88 (25.67-28.14)	
Hypertension				<0.0001
No	1 (reference)	7.39 (7.12-7.66)	46.19 (44.59-47.85)	
Yes	1 (reference)	5.20 (5.03-5.37)	28.72 (27.81-29.67)	
Dyslipidemia				0.0136
No	1 (reference)	5.96 (5.80-6.13)	35.58 (34.61-36.57)	
Yes	1 (reference)	6.47 (6.16-6.78)	37.01 (35.30-38.80)	

aHR adjusted hazard ratio, CI confidence interval

[a] Adjusted for age, income, residential area, diabetes, hypertension, and dyslipidemia

Supplementary Table S5. Subgroup analysis of cardiovascular death by sex, age, income, residential area, and comorbidities

	aHR (95% CI)[a] for cardiovascular death			*p* for interaction
	Localized	Regional	Distant	
Sex				0.6572
Male	1 (reference)	1.25 (1.11-1.41)	1.66 (1.31-2.11)	
Female	1 (reference)	1.33 (1.13-1.58)	1.94 (1.39-2.72)	
Age				<0.0001
30-64	1 (reference)	1.78 (1.41-2.26)	3.93 (2.71-5.69)	
≥65	1 (reference)	1.20 (1.08-1.33)	1.40 (1.11-1.77)	
Income				0.2717
Low	1 (reference)	1.12 (0.91-1.38)	1.93 (1.34-2.79)	
Middle and High	1 (reference)	1.33 (1.19-1.48)	1.68 (1.33-2.11)	
Residential area				0.2893
Metropolitan	1 (reference)	1.16 (0.99-1.35)	1.71 (1.26-2.33)	
Non-metropolitan	1 (reference)	1.36 (1.20-1.53)	1.77 (1.37-2.27)	
Diabetes				0.6380
No	1 (reference)	1.30 (1.16-1.46)	1.85 (1.46-2.34)	
Yes	1 (reference)	1.23 (1.04-1.45)	1.55 (1.10-2.19)	
Hypertension				0.5608
No	1 (reference)	1.37 (1.14-1.64)	1.58 (1.07-2.34)	
Yes	1 (reference)	1.25 (1.11-1.39)	1.80 (1.44-2.26)	
Dyslipidemia				0.2599
No	1 (reference)	1.22 (1.08-1.38)	1.57 (1.22-2.03)	
Yes	1 (reference)	1.37 (1.17-1.59)	2.05 (1.52-2.77)	

aHR adjusted hazard ratio, CI confidence interval

[a] Adjusted for age, income, residential area, diabetes, hypertension, and dyslipidemia

Supplementary Table S6. Subgroup analysis of respiratory death by sex, age, income, residential area, and comorbidities

	aHR (95% CI)[a] for respiratory death			*p* for interaction
	Localized	Regional	Distant	
Sex				0.8046
Male	1 (reference)	1.43 (1.27-1.62)	1.48 (1.10-1.99)	
Female	1 (reference)	1.42 (1.11-1.81)	1.81 (1.05-3.11)	
Age				0.0083
30-64	1 (reference)	1.46 (1.00-2.13)	3.80 (2.09-6.92)	
≥65	1 (reference)	1.43 (1.28-1.60)	1.35 (1.01-1.80)	
Income				0.5448
Low	1 (reference)	1.47 (1.18-1.84)	1.19 (0.67-2.12)	
Middle and High	1 (reference)	1.42 (1.25-1.60)	1.66 (1.25-2.22)	
Residential area				0.4079
Metropolitan	1 (reference)	1.35 (1.13-1.62)	1.81 (1.22-2.70)	
Non-metropolitan	1 (reference)	1.48 (1.29-1.69)	1.40 (0.99-1.96)	
Diabetes				0.5209
No	1 (reference)	1.46 (1.28-1.66)	1.68 (1.25-2.27)	
Yes	1 (reference)	1.36 (1.12-1.66)	1.23 (0.73-2.06)	
Hypertension				0.2365
No	1 (reference)	1.52 (1.28-1.82)	1.97 (1.34-2.90)	
Yes	1 (reference)	1.38 (1.21-1.58)	1.31 (0.93-1.86)	
Dyslipidemia				0.2866
No	1 (reference)	1.35 (1.19-1.54)	1.50 (1.10-2.04)	
Yes	1 (reference)	1.63 (1.34-1.97)	1.66 (1.03-2.66)	

aHR adjusted hazard ratio, CI confidence interval

[a] Adjusted for age, income, residential area, diabetes, hypertension, and dyslipidemia

Supplementary Table S7. Adjusted hazard ratios (aHR) and subdistribution hazard ratios (sHR) for all-cause and cause-specific mortality according to the SEER stage

	Events	Person-years	IR[a]	aHR (95% CI)[b]	sHR (95% CI)[c]
All-cause Death					
Localized	14,647	664,544	26.09	1 (reference)	-
Regional	17,248	166,273	119.61	4.31 (4.22-4.40)	-
Distant	16,588	28,024	810.87	24.73 (24.22-25.26)	-
Stomach Cancer Death					
Localized	6938	664,544	10.44	1 (reference)	1 (reference)
Regional	16,515	166,273	99.32	8.70 (8.46-8.94)	8.56 (8.32-8.81)
Distant	21,652	28,024	772.62	51.67 (50.24-53.14)	43.79 (42.52-45.10)
Cancer Death					
Localized	10,733	664,544	16.15	1 (reference)	1 (reference)
Regional	17,689	166,273	106.39	6.08 (5.94-6.23)	6.32 (6.16-6.48)
Distant	22,259	28,024	794.28	35.97 (35.11-36.85)	34.01 (33.13-34.92)
Cardiovascular Death					
Localized	1766	664,544	2.66	1 (reference)	1 (reference)
Regional	555	166,273	3.34	1.28 (1.16-1.41)	0.83 (0.75-0.92)
Distant	111	28,024	3.96	1.74 (1.43-2.12)	0.31 (0.25-0.38)
Respiratory Death					
Localized	1306	664,544	1.97	1 (reference)	1 (reference)
Regional	452	166,273	2.72	1.43 (1.28-1.59)	0.85 (0.76-0.95)
Distant	62	28,024	2.21	1.54 (1.19-2.00)	0.21 (0.16-0.27)

SEER surveillance, epidemiology, and end results, aHR adjusted hazard ratio, sHR subdistribution hazard ratio,CI confidence interval, IR incidence rate

[a] Incidence rate: Per 1,000 person-years

[b] adjusted hazard ratio from multivariable Cox proportional hazards model adjusted for sex, age, income, residential area, diabetes, hypertension, and dyslipidemia

[c] adjusted subdistribution hazard ratio from multivariable Fine-Gray model adjusted for sex, age, income, residential area, diabetes, hypertension, and dyslipidemia

Supplementary Table S8. Sensitivity analysis excluding patients who died within 90 days of diagnosis: All-cause and cause-specific mortality according to the SEER stage

	Events	Person-years	IR[a]	HR (95% CI)			
				Model 1[b]	Model 2[c]	Model 3[d]	Model 4[e]
All-cause Death							
Localized	14,647	630,415	23.23	1 (reference)	1 (reference)	1 (reference)	1 (reference)
Regional	17,248	153,424	112.42	4.65 (4.55-4.75)	4.66 (4.56-4.76)	4.64 (4.54-4.74)	4.59 (4.49-4.69)
Distant	16,588	23,061	719.30	24.08 (23.53-24.64)	26.53 (25.92-27.15)	26.42 (25.81-27.04)	26.07 (25.47-26.69)
Stomach Cancer Death							
Localized	5526	630,415	8.77	1 (reference)	1 (reference)	1 (reference)	1 (reference)
Regional	14,349	153,424	93.53	10.00 (9.69-10.31)	10.00 (9.69-10.31)	9.97 (9.67-10.28)	9.84 (9.54-10.15)
Distant	15,970	23,061	692.50	54.18 (52.51-55.91)	58.58 (56.76-60.45)	58.41 (56.61-60.28)	57.44 (55.66-59.28)
Cancer Death							
Localized	8488	630,415	13.46	1 (reference)	1 (reference)	1 (reference)	1 (reference)
Regional	15,263	153,424	99.48	6.99 (6.81-7.18)	7.00 (6.82-7.19)	6.98 (6.80-7.17)	6.88 (6.70-7.07)
Distant	16,286	23,061	706.20	37.78 (36.77-38.82)	41.08 (39.98-42.22)	40.96 (39.86-42.09)	40.26 (39.18-41.37)
Cardiovascular Death							
Localized	1645	630,415	2.61	1 (reference)	1 (reference)	1 (reference)	1 (reference)
Regional	495	153,424	3.23	1.26 (1.14-1.39)	1.25 (1.13-1.38)	1.25 (1.13-1.38)	1.27 (1.14-1.40)
Distant	68	23,061	2.95	1.26 (0.99-1.61)	1.46 (1.14-1.87)	1.45 (1.14-1.86)	1.50 (1.17-1.92)
Respiratory Death							
Localized	1221	630,415	1.94	1 (reference)	1 (reference)	1 (reference)	1 (reference)
Regional	407	153,424	2.65	1.42 (1.27-1.58)	1.42 (1.27-1.59)	1.41 (1.26-1.58)	1.41 (1.26-1.58)
Distant	42	23,061	1.82	1.18 (0.86-1.61)	1.39 (1.02-1.90)	1.37 (1.01-1.88)	1.37 (1.01-1.88)

SEER surveillance, epidemiology, and end results, HR hazard ratio, CI confidence interval, IR incidence rate

[a] Incidence rate: Per 1,000 person-years

[b] Model 1: Non-adjusted

[c] Model 2: Adjusted for sex and age

[d] Model 3: Adjusted for sex, age, income, and residential area

[e] Model 4: Adjusted for sex, age, income, residential area, diabetes, hypertension, and dyslipidemia

Supplementary Table S9. Sensitivity analysis for additional adjustment for smoking, alcohol use, and body mass index: All-cause and cause-specific mortality according to the SEER stage

	No.	Events	Person-years	IR[a]	aHR (95% CI)[b]
All-cause Death					
Localized	74,153	5097	303,348	16.80	1 (reference)
Regional	17,034	5597	58,269	96.05	5.44 (5.23-5.65)
Distant	7482	6629	9338	709.92	38.91 (37.44-40.43)
Stomach Cancer Death					
Localized	74,153	1757	303,348	5.79	1 (reference)
Regional	17,034	4688	58,269	80.45	13.04 (12.34-13.77)
Distant	7482	6356	9338	680.68	95.75 (90.68-101.10)
Cancer Death					
Localized	74,153	2932	303,348	9.67	1 (reference)
Regional	17,034	4984	58,269	85.53	8.36 (7.99-8.76)
Distant	7482	6515	9338	697.71	62.03 (59.28-64.91)
Cardiovascular Death					
Localized	74,153	570	303,348	1.88	1 (reference)
Regional	17,034	151	58,269	2.59	1.34 (1.12-1.61)
Distant	7482	28	9338	3.00	1.98 (1.35-2.92)
Respiratory Death					
Localized	74,153	408	303,348	1.34	1 (reference)
Regional	17,034	122	58,269	2.09	1.52 (1.24-1.87)
Distant	7482	14	9338	1.50	1.57 (0.92-2.69)

SEER surveillance, epidemiology, and end results, aHR adjusted hazard ratio, CI confidence interval, IR incidence rate

[a] Incidence rate: Per 1,000 person-years

[b] Adjusted for sex, age, income, residential area, diabetes, hypertension, dyslipidemia, smoking, alcohol use, and body mass index (BMI)
